# Brushfield spots and Wölfflin nodules unveiled in dark irides using near-infrared light

**DOI:** 10.1038/s41598-018-36348-6

**Published:** 2018-12-21

**Authors:** Lavinia Postolache, Cameron F. Parsa

**Affiliations:** 1 Queen Fabiola University Children’s Hospital, Université Libre de Bruxelles, Brussels, Belgium; 2 Erasmus Hospital, Université Libre de Bruxelles, Brussels, Belgium; 3 Quinze-Vingts National Eye Hospital, Sorbonne University, Paris, France

## Abstract

Wölfflin nodules and Brushfield spots were described essentially in light colored irides. The purpose of our study is to determine if these iris features are also present in dark irides, hidden by melanin granules of the anterior leaf of the iris. We examined iris images, taken with standard visible white, as well as with near-infrared light of children with Down syndrome and without. Using white light, Brushfield spots were seen in 21% of children with Down syndrome, and Wölfflin nodules in 12% of controls (p < 0.001), all noted in those with lightly colored irides. Brushfield spots were detected in 67% of children with Down syndrome using near-infrared light compared to 21% using white light (p < 0.001). Wölfflin nodules were detected in 19% of controls using near-infrared light compared to 12% using white light. Peripheral iris thinning was present in 63% of children with Down syndrome but in only 23% of those without (p = 0.001). Contraction furrows were less frequent in children with Down syndrome (16%) compared to controls (74%)(p < 0.001). Near-infrared light unveils Brushfield spots and Wölfflin nodules in dark irides. Clearing this discrepancy should assist in the elucidation of their pathophysiologic origin. A high prevalence of peripheral iris thinning is also present in children with Down syndrome along with a heretofore unreported reduction in iris contraction furrows.

## Introduction

In 1924, the English psychiatrist Thomas Brushfield described white spots of varying sizes encircling the mid-periphery of only lightly colored irides in children with Down syndrome. The easily visible iris nodules described quickly became known as “Brushfield spots” and appreciated as a diagnostic indicator for the anomaly then called mongolism^1^, often referred to now as trisomy 21. Somewhat similar, but smaller and more peripherally located iris nodules termed Wölfflin nodules^2^, had also been recognized in approximately 10% of individuals with lightly colored irides. Prior to the advent of karyotyping, Brushfield spots were widely discussed and taught in medical schools as an aid in the diagnosis of Down syndrome. Today, the clinical application has effectively diminished to the point of being of mostly historical interest. Despite the determination of the defining triallelic chromosomal feature of Down syndrome by Gautier and colleagues in 1959 following the introduction of karyotyping^3^, interest in the significance of these recognizable iris features persists. Such iris spots or nodules, however, have heretofore essentially only been described in individuals with lightly colored irides^1,2,4–9^ with the discrepancy, as well as origin, effectively unaccounted for. In the course of iris photography, we employed both standard visible white as well as near-infrared light to better determine and characterize if such iris features were also present in darker colored irides. We also evaluated the differences between the peripheral thinning and contraction furrows in individuals with and without Down syndrome.

## Results

### Study Demographics

Study population characteristics are summarized in Table 1. There were 43 children with Down syndrome, mean age 7.2 ± 3.8 years and 43 controls with mean age 8.8 ± 2.9 years.

**Table 1:** Demographic data and distribution of iris color in Down syndrome versus controls.

		Down syndrome N = 43	Control N = 43
Age (SD)		7.2 ± 3.8 years	8.8 ± 2.9 years
Sex		30 boys	21 boys
Race	White	38	39
	Black	5	4
Iris color	Blue	8	7
	Hazel	4	4
	Brown	31	32

### Results of imaging using standard white light

**Figure 1:**
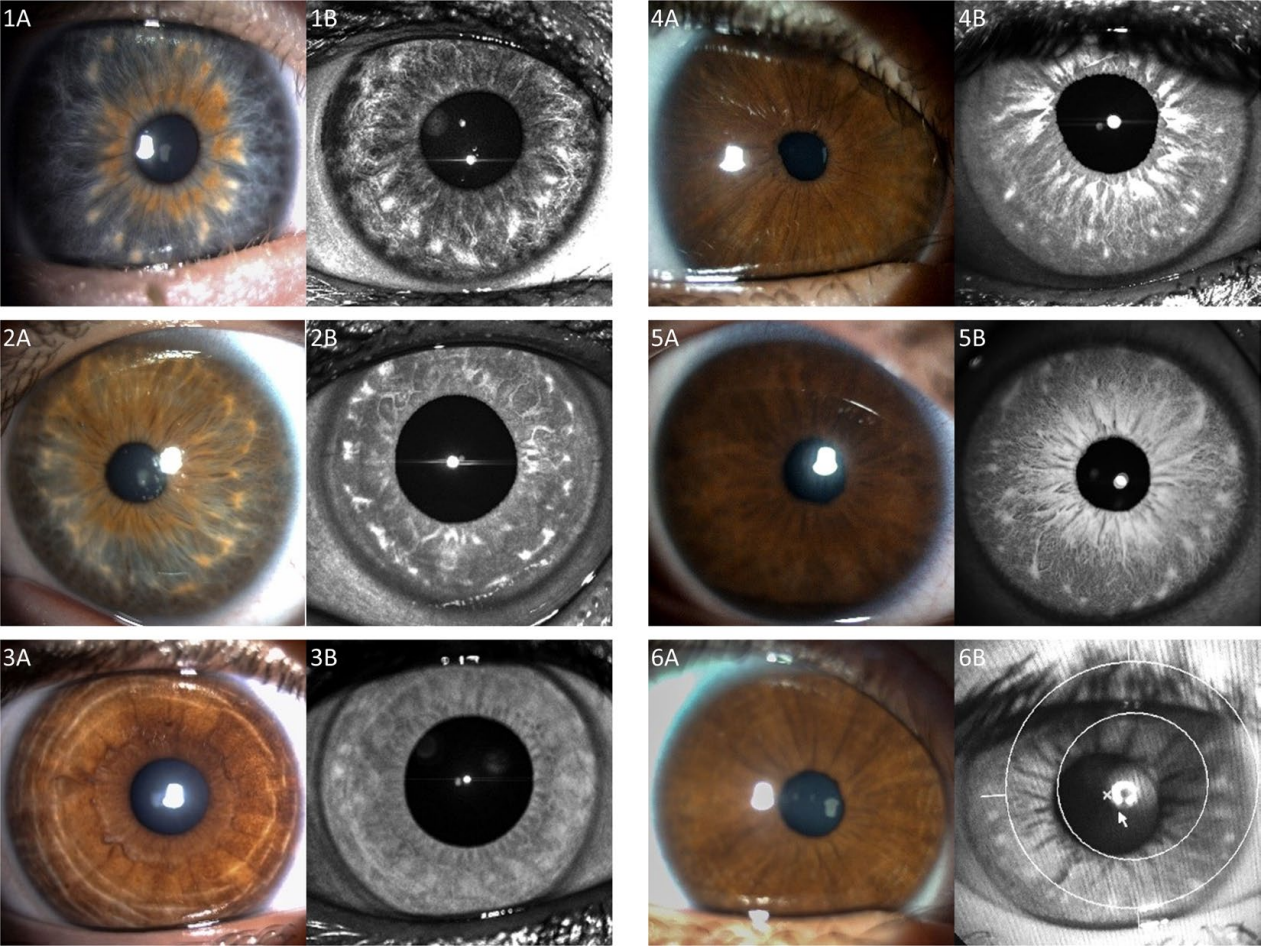
Visibility of Brushfield spots and Wölfflin nodules under white (Column A), versus near-infrared (Column B) illumination. (**1A**) Brushfield spots and extensive iris thinning peripheral to these spots is seen with standard white light in a child with Down syndrome and blue irides. (**1B**) Same Brushfield spots noted with 820 nm wavelength near-infrared photography. (**2A**) Brushfield spots seen using white light in a patient with Down syndrome and hazel irides. (**2B**) Same Brushfield spots noted with 820 nm wavelength near-infrared photography. (**3A**) Standard white light iris photography fails to reveal any characteristic iris spots or nodules in a control. (**3B**) 820 nm wavelength near-infrared photography discloses Wölfflin nodules. (**4A**, **5A**) In these brown-eyed children with Down syndrome, no Brushfield spots are apparent using standard visible white light. (**4B**,**5B**) In the same children, Brushfield spots appear when using 820 nm wavelength near-infrared light. one may also readily recognize here a paucity of iris contraction furrows in children with Down syndrome. (**6A**) Child with Down syndrome and brown irides with no apparent spots using standard visible white light. (**6B**) Brushfield spots in the same child become detectable using the fundus camera with a 650–735 nm wavelength barrier filter for near-infrared photography. Compared with the brown irides noted amongst most patient controls (**3A**) contraction furrows were lacking in the brown irides of children with Down syndrome (**4A**,**5A**) or were almost imperceptible (**6A**).

Brushfield spots were observed in only 9 of the 43 children with Down syndrome (21%) using standard visible white light. All of these were noted only in those with light colored irides (i.e., blue or hazel). Figure 1 illustrates examples of Brushfield spots visible with white light in lightly colored eyes (Fig. 1 1A,2A).

**Figure 2:**
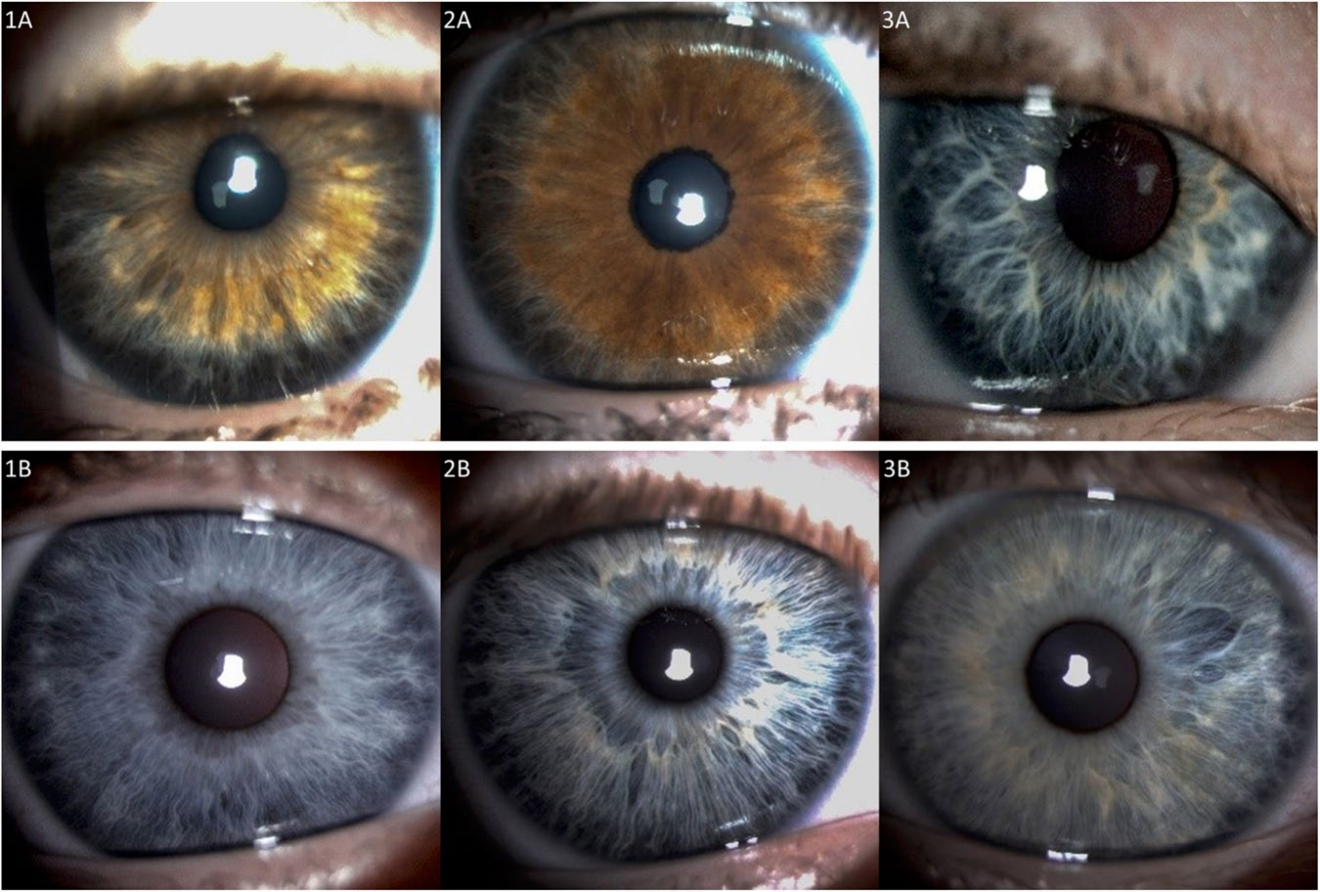
Peripherally thinned irides in children with Down syndrome (Row A) versus controls (Row B). (Row A) Extensive peripheral iris thinning and absence of contraction furrows in light brown, hazel, and blue irides in children with Down syndrome. Brushfield spots were located between normal and thinned iris. (Row B) Less extensive iris thinning in control children with blue irides, which may not be present in those with darker irides (not shown). (**1B**,**3B**) Contraction furrows are present in two children, as are Wölfflin nodules, found at the demarcation between normal and thinned iris.

Wölfflin nodules were observed in only five control patients without Down syndrome (12%) using visible white light (Fig. 2 1B,3B, Supplemental Fig. 1 5A,6A and 11A).

The results as a function of iris color are summarized in Table 2, with individual level results shown in Supplementary Tables S1 and S2.

**Table 2:** Brushfield spots and Wölfflin nodules based on white versus near-infrared light.

Iris color	Down syndrome		Control		p-value
	White light	Near-infrared light	White light	Near-infrared light	
Blue	7	7	3	3	
Hazel	2	4	1	1	
Brown	0	18	1	4	
All	9	29	5	8	<0.001*

*p-value for both all detected Brushfield spots in Down syndrome versus Wölfflin nodules in controls and combined white versus near-infrared based light detection of iris spots and nodules.

### Results of imaging using near-infrared light

We noted the presence of Brushfield spots in another twenty of the children with Down syndrome, all with darker colored eyes, for detection in a total of 29/43 (67%). Figure 1 provides illustrative examples obtained from children with brown eyes and Down syndrome using visible white light (Fig. 1 4A–6A) but in which such spots become visible using near-infrared at 820 nm (Fig. 1 4B,5B) and at 650–735 nm wavelength illumination (Fig. 1 6B and Supplemental Fig. 1 Columns B). With near-infrared illumination, iris nodules were also detected in another three of the children possessing dark irides and without Down syndrome, for a total frequency of detection of 19%, compared to 67% in Down syndrome patients (p < 0.001). Figure 1 demonstrates a brown iris with no apparent Wölfflin nodules using white light (Fig. 1 3A), but with some Wölfflin nodules rendered visible by near-infrared 820 nm wavelength illumination (Fig. 1 3B).

In patients in whom Brushfield spots or Wölfflin nodules were already visible using white light, we found the same number and distribution using near-infrared light with no additional spots or nodules noted.

However, disclosure of Brushfield spots in children with dark irides, otherwise undetectable with standard white light, was discovered using near-infrared illumination. The ability to detect such features in White children with Down syndrome and dark irides rose from 0% to 58% (p < 0.001).

No difference was noted regarding the number or the extent of Brushfield spots or Wölfflin nodules between iris images taken with the confocal laser (820 nm wavelength) compared to images provided by the non-mydriatic fundus camera (650–735 nm wavelength). Direct confocal laser imagery taken using 820 nm wavelength illumination did allow, however, more contrasted visualization of the individual iris spots or nodules.

Nonetheless, whether using visible or the near-infrared light at 650–735 nm wavelengths, we were still unable to detect Brushfield spots or Wölfflin nodules in the darkest irides of the nine Black children. Supplemental Fig. 1 10A,10B shows the aspect of a dark brown iris in a Black child with Down syndrome in white light and in near-infrared light respectively.

### Assessment of iris peripheral thinning and contraction furrows

Twenty-seven out of the 43 children with Down syndrome (63%) compared to 10 out of the 43 controls (23%) (p < 0.001) presented with various degrees of peripheral iris thinning, detected via visible white light as well as near-infrared light (Table 3).

**Table 3:** Peripheral iris thinning and contraction furrows in Down syndrome versus controls.

Iris color	Down syndrome		Control		p-value
	Iris thinning	Iris contraction furrows	Iris thinning	Iris contraction furrows	
Blue	8	0	6	4	
Hazel	4	0	3	3	
Brown	15	7	1	25	
All	27	7	10	32	<0.001*

*p-value for both all iris thinning and contraction furrows in Down syndrome versus control.

However, the area of such thinning was far less extensive in those without Down syndrome. Figure 2 provides examples of peripheral iris thinning in children with Down syndrome (Row A) and without (Row B). The great majority of children with Down syndrome who had peripheral iris thinning also had Brushfield spots (22/27). A similar finding was also noted in the children without Down syndrome; five out of the ten children who had peripheral iris thinning also exhibited Wölfflin nodules.

Iris contraction furrows were present in 32 out of 43 (74%) of children without Down syndrome, but present in only 7 out of 43 children with Down syndrome (16%)(p < 0.001) (Table 3). In only two of these did the contraction furrows coexist with a certain degree of iris peripheral thinning (Supplementary Tables S1 and S2).

## Discussion

Whereas Brushfield spots are widely reported in individuals with Down syndrome and Wölfflin nodules are estimated to be present in up to 10% of the normal population, their pathophysiologic origin nonetheless remains unexplained. Histopathological examination reveals them to consist of a condensation or mound of collagen tissue^4,9,10^. Wölfflin nodules may be considered a variant of smaller spots noted more peripherally in the lightly colored irides of some individuals without Down syndrome. Nevertheless, the frequent incidence of the larger variants in Down syndrome, as well as their occurrence reported essentially only in lightly colored irides^1,2,4–9^ has remained enigmatic. In 1961, using a special flash illumination and high magnification photographic system, Donaldson was able to detect a prevalence of Brushfield spots and/or iris speckling in 77% of individuals with Down syndrome with brown irides, and a prevalence of Wölfflin nodules in 13% of controls with brown irides^11^, both figures remarkably close to what we were able to disclose using infrared wavelengths. Falls also believed the specific iris features to be less conspicuous in darker eyes^12^, likely hidden by the higher density and number of melanin granules in the anterior-border layer of darker colored irides^13,14^.

Melanin is relatively transparent to infrared wavelengths^15^ and such easily tolerated iris illumination has been employed for identity screening and confirmation in the general population^16,17^, as well as for iris-crypt-based video-oculography to measure ocular torsion^18^ .Although suggested previously^13^, it had not been applied as a means to investigate the iris itself such as for the visualization and study of Wöllflin nodules or Brushfield spots. In those with lightly colored eyes with iris spots or nodules already visible in standard white light, we did not detect additional spots or nodules using near-infrared wavelengths.

However, in those with hazel and brown irides, the prevalence of detectable spots increased dramatically when using near infrared as opposed to visible light. Using visible light, Brushfield spots were only seen in 2/4 (50%) of those with hazel irides, but in all four (100%) with infrared light. In those with brown irides, no spots were seen when using visible light, but 18 out of 31(58%) had Brushfield spots detectable using near-infrared light. Hence, the overall percentage of children with Down syndrome presenting with Brushfield spots tripled, from only 21% under standard white light conditions, to 67% using near-infrared illumination. Infrared light observation also increased the detectability of Wölfflin nodules in children without Down syndrome from 12% in white light, to 19% using near-infrared light.

As alternatively demonstrated by Donaldson^11^, it appears that the visibility of Brushfield spots and of Wölfflin nodules in eyes of different color can be attributed in part to a “camouflaging” effect of overlying melanocytes. The fact that with either the intense white light illumination techniques used by Donaldson, or with the easily tolerated near-infrared wavelengths now employed, the number of subjects with dark irides and detectable iris spots still remains slightly inferior to that of those with lighter colored irides indicates that other factors might also be at play. Nonetheless, the marked narrowing of apparent disparities between light and dark irides allows for more straightforward pathophysiologic mechanisms to be proposed.

Thinning of the peripheral portion of the iris, present in a minority (8–9%) of the general population^4,11^ may, on the other hand, be noted in the majority (81–95%) of individuals with Down syndrome^11,19^. In our study, we noted thinning in 23% of presumed normal children, and in 63% of children with Down syndrome. The thinner, hypoplastic areas appeared as a peripheral ring of darker-colored iris using near-infrared light, but with a lower resolution of iris details vis-à-vis slit lamp examination with standard white light. Atrophic iris thinning is a feature of the natural aging process in the general population, attributed to the biological decrease in tissue vascularity^4,19^. Skeller, in 1951, suggested that reduced vascular development *in utero* might be a cause for hypoplastic iris development in Down syndrome^20^. As angiogenesis^21,22^ is now understood to be diminished in Down syndrome^23–25^ such arguments become more plausible. We also noted fewer contraction furrows in the irides of patients with Down syndrome. This may also be due to an underdevelopment of peripheral portions of the radial iris dilator muscles.

While Brushfield spots had often been described to represent a relative accumulation of stromal fibres “surrounded” by iris hypoplasia^10,11^, we found variable levels of iris hypoplasia mainly on the outer periphery of spots. Brushfield spots were also often found to be located along a demarcation line between apparently normal and hypoplastic iris. Inspection of published photographs^4,5,11,20^ tends to corroborate this observation. The accumulated data should allow for more straightforward pathophysiologic mechanisms to be proposed explaining their aetiology.

### Study limitations and possible biases

The fact that observers were not blind to subject status could create a potential bias in iris nodule detection. The exclusion of younger or less cooperative children with Down syndrome who were not able to cooperate sufficiently for iris imaging, as well as the selection of the controls, could also potentially generate biases.

Fewer normal children without Down syndrome had images of the iris using higher resolution confocal scanning laser ophthalmoscope combined with optical coherence tomography. However, analysis of the images already available from both imaging systems did not detect any difference in the number and disposition of iris nodules or spots detected using the two different systems.

## Methods

### Subjects

During a six-month period (September 2016 - March 2017), sixty patients with Down syndrome each underwent a multidisciplinary work-up, including complete ophthalmological examinations at Queen Fabiola University Children’s Hospital in Brussels, Belgium. Iris imaging using white, standard light and near-infrared light was performed in forty-three patients. Due to the inability for some patients to cooperate sufficiently, iris photography was not performed in seventeen out of the sixty patients with Down syndrome (28%) who were hence excluded. Forty-three consecutive, otherwise healthy, non-hospitalized children without Down syndrome, seen for refractive or strabismus issues except for one patient with congenital motor nystagmus, had similar photographic documentation within the same time period and were selected as a control group.

Informed consent was obtained from all children and/or their parents, with Institutional Review Board and Institutional Ethics Committee of Queen Fabiola University Children’s Hospital approval (CHE No. 19/17). All examinations were performed in accordance with the principles and tenets of the Declaration of Helsinki.

### Imaging

All subjects were examined using slit-lamp biomicroscopy (Topcon SL-D701 TR, Tokyo, Japan) using standard white light (LED 3A, 10W, Topcon, Tokyo, Japan) to detect specific iris particularities such as Brushfield spots, Wölfflin nodules, peripheral iris thinning and contraction furrows. Iris thinning was noted as a peripheral ring of discoloration, at the level of the iris root that appeared as a dark background in lightly colored irides, and as a light discoloration in darkly colored irides, occasionally showing blood columns visible through bared vessels. Photographs were obtained with 16x and 40x magnification using a digital DC-4 photographic module (Topcon, Tokyo, Japan). A non-mydriatic fundus camera (Visucam R 500, Zeiss, Jena, Germany) was used which projects infrared light through a 650–735 nm barrier filter in order to illuminate the anterior segment at maximum intensity and assist in patient positioning. The camera itself, however, does not allow for direct infrared photography because of the simultaneous triggering of a xenon flash at the moment of image acquisition. Hence, an 8-megapixel *f/2*.*2* aperture iPhone 6 camera (Apple Inc., Cupertino, California) was used to obtain images of the screen monitor linked to the fundus camera displaying the iris illuminated by infrared light.

In addition, seventeen patients with Down syndrome and six non-triallelic children who were able to go to the adult unit also underwent anterior segment examination and imaging using a confocal scanning laser ophthalmoscope combined with optical coherence tomography (Heidelberg Spectralis R Tracking Laser Tomography, Heidelberg, Germany). This device is equipped with an 820 nm wavelength laser diode that permits direct infrared illumination as well as photography.

### Assessment of images

We analysed iris images, taken in both visible and near-infrared light conditions, for the presence of Brushfield spots or Wölfflin nodules using three different categories: no iris spots, spots within less than 180° of the iris diaphragm circumference, or spots within more than 180° of the iris diaphragm circumference. We also noted any peripheral thinning of the iris and the presence of iris contraction furrows.

Wölfflin nodules were defined as white or yellowish iris nodules disposed in a circular pattern, in the ciliary portion of the iris, at variable distances from the periphery. Brushfield were of similar appearance, but often bigger and placed closer to the pupil margin compared to Wölfflin nodules.

The peripheral iris thinning was noted as areas of stromal paucity “ring like” at the iris root with variable extension. High magnification biomicroscopy with visible white light reveals further details such as bared blood vessels in areas of iris thinning. Iris thinning was absent from the more central portions of the iris diaphragm. No defect of the posterior iris pigmentary epithelium could be noted in any area via transpupillary iris illumination. In white-standard light the peripheral iris thinning appeared as a darker ring in lightly colored irides or as a discolored ring in darker/brown irides.

In near-infrared illumination, the thinned areas appeared darker than the rest of the iris stroma.

### Statistical analysis

Descriptive statistics for categorical data are presented in frequencies and numeric data as mean values, with standard deviations. Analyses include both eyes per participant.

Given similar distribution of age, race, iris colors, multivariate-based adjustments were not required to account for unequal distribution of key variables. Statistical significance for differences in observed dichotomous outcomes such as presence of iris nodules (Brushfield spots in participants with Down syndrome, and Wölfflin nodules in those without Down syndrome), were calculated using Fisher’s exact test with a significance level alpha set at 0.05, based on 2-tailed *P* values.

## Electronic supplementary material

Dataset 1

## Data Availability

All data were available upon request. In case of any further information, Dr. Lavinia Postolache can be contacted at Lavinia.Postolache@ulb.ac.be.
