# Supplementary material for: Brushfield spots and Wölfflin nodules unveiled in dark irides using near-infrared light
Source: Sci Rep. 2018 Dec 21;8:18040. doi: 10.1038/s41598-018-36348-6 (PMC6303377; doi:10.1038/s41598-018-36348-6)

**Supplementary Information File**

# **Brushfield spots and Wölfflin nodules unveiled in dark irides using near-infrared light**

# **Lavinia Postolache*^1^ and Cameron F. Parsa^2,3^**

^1^ Queen Fabiola University Children’s Hospital, Université Libre de Bruxelles,

Brussels, Belgium

^2^ Erasmus Hospital, Université Libre de Bruxelles, Brussels, Belgium

^3^ Quinze-Vingts National Eye Hospital, Sorbonne University, Paris, France

Correspondence address: Lavinia Postolache, M.D., Service d’Ophtalmologie, Hôpital Erasme, 808, Route de Lennik, 1070, Brussels, Belgium Tel: +32 25553114, Fax: +32 25556737, Email: Lavinia.Postolache@ulb.ac.be

**SUPPLEMENTAL TABLE 1**. Individual level characteristics and findings in children with Down syndrome

| Patient number | Age | Sex | Race | Iris color | Brushfield spots using visible light | Brushfield spots on <180° iris using near-infrared light | Presence of Brushfield spots on >180° iris using near-infrared light | Peripheral iris thinning | Iris contraction furrows |
| --- | --- | --- | --- | --- | --- | --- | --- | --- | --- |
| 1 | 4y8m | M | Black | Brown |  |  |  | x |  |
| 2 | 6y8m | M | White | Brown |  |  | x | x |  |
| 3 | 8y1m | M | White | Brown |  |  |  |  | x |
| 4 | 10y7m | M | White | Brown |  | x |  |  | x |
| 5 | 7y8m | M | White | Brown |  |  | x | x |  |
| 6 | 2y10m | M | White | Hazel | x |  | x | x |  |
| 7 | 7y | F | White | Brown |  |  | x |  | x |
| 8 | 1y11m | F | White | Brown |  |  | x | x |  |
| 9 | 4y7m | F | White | Brown |  |  | x |  |  |
| 10 | 5y5m | M | White | Blue | x |  | x | x |  |
| 11 | 4y8m | M | White | Brown |  |  | x | x |  |
| 12 | 6y6m | M | White | Brown |  |  | x |  |  |
| 13 | 4y8m | F | White | Brown |  |  |  |  |  |
| 14 | 11y7m | M | White | Brown |  |  | x | x |  |
| 15 | 5y10m | M | White | Brown |  | x |  | x |  |
| 16 | 5y9m | M | White | Brown |  |  |  |  |  |
| 17 | 2y | F | White | Blue | x |  | x | x |  |
| 18 | 10y | M | White | Blue | x |  | x | x |  |
| 19 | 10y5m | M | White | Brown |  | x |  |  |  |
| 20 | 3y9m | M | White | Hazel | x |  | x | x |  |
| 21 | 6y7m | F | White | Hazel |  |  | x | x |  |
| 22 | 0y7m | M | White | Blue -gray |  |  |  | x |  |
| 23 | 15y3m | F | White | Brown |  |  | x |  | x |
| 24 | 12y7m | M | White | Hazel |  |  | x | x |  |
| 25 | 7y5m | M | White | Brown |  |  |  | x | x |
| 26 | 7y10m | M | White | Brown |  |  |  |  |  |
| 27 | 13y4m | M | White | Brown |  | x |  | x |  |
| 28 | 6y1m | M | White | Brown |  |  | x | x |  |
| 29 | 11y5m | M | White | Brown |  | x |  |  |  |
| 30 | 13y3m | M | White | Blue | x |  | x | x |  |
| 31 | 6y6m | F | White | Brown |  |  | x |  | x |
| 32 | 5y3m | F | Black | Brown |  |  |  |  |  |
| 33 | 3y6m | F | White | Brown |  |  |  |  |  |
| 34 | 7y8m | F | White | Brown |  |  |  | x |  |
| 35 | 9y1m | M | White | Brown |  |  | x | x |  |
| 36 | 12y3m | F | White | Blue | x |  | x | x |  |
| 37 | 15y6m | M | Black | Brown |  |  |  | x |  |
| 38 | 2y1m | M | White | Brown |  |  |  |  |  |
| 39 | 3y1m | M | White | Blue | x |  | x | x |  |
| 40 | 9y1m | F | White | Brown |  |  | x | x |  |
| 41 | 4y10m | M | White | Blue | x |  | x | x |  |
| 42 | 10y3m | M | Black | Brown |  |  |  | x | x |
| 43 | 5y3m | M | Black | Brown |  |  |  |  |  |

**SUPPLEMENTAL TABLE 2.** Individual level characteristics and findings in children without Down syndrome

| Patient number | Age | Sex | Race | Iris color | Wölfflin nodules using visible light | Wölfflin nodules on <180° of iris diaphragm using near- infrared light | Wölfflin nodules on >180° of iris diaphragm using near- infrared light | Peripheral iris thinning | Iris contraction furrows |
| --- | --- | --- | --- | --- | --- | --- | --- | --- | --- |
| 1 | 7y6m | M | White | Brown |  |  |  |  | x |
| 2 | 11y8m | M | White | Brown |  |  |  |  | x |
| 3 | 9y5m | M | White | Brown |  |  |  |  | x |
| 4 | 11y8m | F | White | Brown |  | x |  |  | x |
| 5 | 6y1m | M | White | Brown |  |  |  |  | x |
| 6 | 3y9m | M | White | Brown |  | x |  |  |  |
| 7 | 10y7m | F | White | Brown |  |  |  |  | x |
| 8 | 7y11m | M | White | Hazel | x |  | x | x |  |
| 9 | 15y | F | Black | Brown |  |  |  |  |  |
| 10 | 5y8m | F | White | Brown |  |  |  |  | x |
| 11 | 11y7m | F | Black | Brown |  |  |  |  | x |
| 12 | 8y9m | M | White | Brown |  |  |  |  | x |
| 13 | 6y11m | M | White | Brown |  |  |  |  | x |
| 14 | 12y7m | M | White | Brown |  |  |  |  | x |
| 15 | 6y11m | F | White | Brown |  |  |  |  | x |
| 16 | 6y6m | F | White | Blue |  |  |  | x |  |
| 17 | 6y6m | M | White | Blue |  |  |  | x |  |
| 18 | 7y5m | F | Black | Brown |  |  |  |  | x |
| 19 | 4y11m | F | White | Hazel |  |  |  |  | x |
| 20 | 7y3m | F | White | Hazel |  |  |  |  | x |
| 21 | 5y2m | F | White | Blue |  |  |  | x |  |
| 22 | 12y7m | M | White | Brown |  | x |  |  | x |
| 23 | 8y3m | F | White | Brown |  |  |  |  | x |
| 24 | 9y9m | F | White | Brown |  |  |  |  |  |
| 25 | 6y10m | M | White | Blue |  |  |  | x | x |
| 26 | 10y3m | F | White | Brown |  |  |  |  | x |
| 27 | 12y7m | F | White | Brown |  |  |  |  | x |
| 28 | 10y5m | F | White | Brown |  |  |  |  | x |
| 29 | 15y5m | F | White | Brown |  |  |  |  |  |
| 30 | 5y3m | M | White | Brown | x |  | x | x | x |
| 31 | 12y7m | M | White | Brown |  |  |  |  |  |
| 32 | 5y2m | M | White | Brown |  |  |  |  | x |
| 33 | 11y5m | M | White | Brown |  |  |  |  | x |
| 34 | 7y7m | F | Black | Brown |  |  |  |  | x |
| 35 | 8y1m | F | White | Hazel |  |  |  | x | x |
| 36 | 10y4m | M | White | Brown |  |  |  |  | x |
| 37 | 5y | M | White | Brown |  |  |  |  | x |
| 38 | 10y4m | M | White | Brown |  |  |  |  |  |
| 39 | 4y8m | M | White | Blue | x |  | x | x | x |
| 40 | 7y9m | F | White | Blue | x |  | x | x | x |
| 41 | 9y5m | F | White | Blue | x |  | x | x | x |
| 42 | 11y1m | M | White | Brown |  |  |  |  |  |
| 43 | 9y2m | F | White | Brown |  |  |  |  | x |

**Supplemental FIGURE 1**

**Legend**

**FIGURE 1.** **(Supplemental)** Visibility of Brushfield spots and Wölfflin nodules with standard white, (Columns A), versus near-infrared 650-735 nm (Columns B) wavelength illumination.

(1A and 2A) Brushfield spots noted in standard white light in two children with Down syndrome and blue irides. (1B and 2B) Brushfield spots using the 650-735 nm barrier filter of the non-mydriatic fundus camera in the same children. (3A and 3B) Brushfield spots visible in a child with Down syndrome and hazel irides in white, and near-infrared light, respectively.

(4A and 7A) illustrate brown irides in two children with Down syndrome. Broad rings of peripheral iris thinning are noted using standard white light in areas often described as “discolored”. Brushfield spots, invisible under standard illumination, are revealed by near-infrared light at the junction between thinned and normal areas of iris (darker peripheral ring vis-à-vis more central zone) (4B and 7B). (8A and 9A). Brushfield spots undisclosed in brown irides of two children with Down syndrome using white light, became visible using near-infrared light (8B and 9B).

(10A) Brown iris in a Black child with Down syndrome. (10B) Despite the near-infrared illumination, no Brushfield spots could be noted.

(5A and 5B) Wölfflin nodules in a normal child with light brown irides visible in standard white, as well as in near-infrared, light (white arrows). (6A) Wölfflin nodules and peripheral iris thinning in a normal child with blue irides. (6B) Wölfflin nodules and peripheral iris thinning noted with near-infrared 650-735 nm wavelength illumination. (11 A) Hazel-colored iris with peripheral thinning and Wölfflin nodules seen in a normal child under standard white light. In this instance, the area of iris thinning is broad and the Wölfflin nodules located closer to the pupillary margin. (11B) Appearance in the same child using near-infrared illumination. (12A and 12 B) Brown iris of the child’s sibling viewed in standard white, as well as near-infrared, illumination.


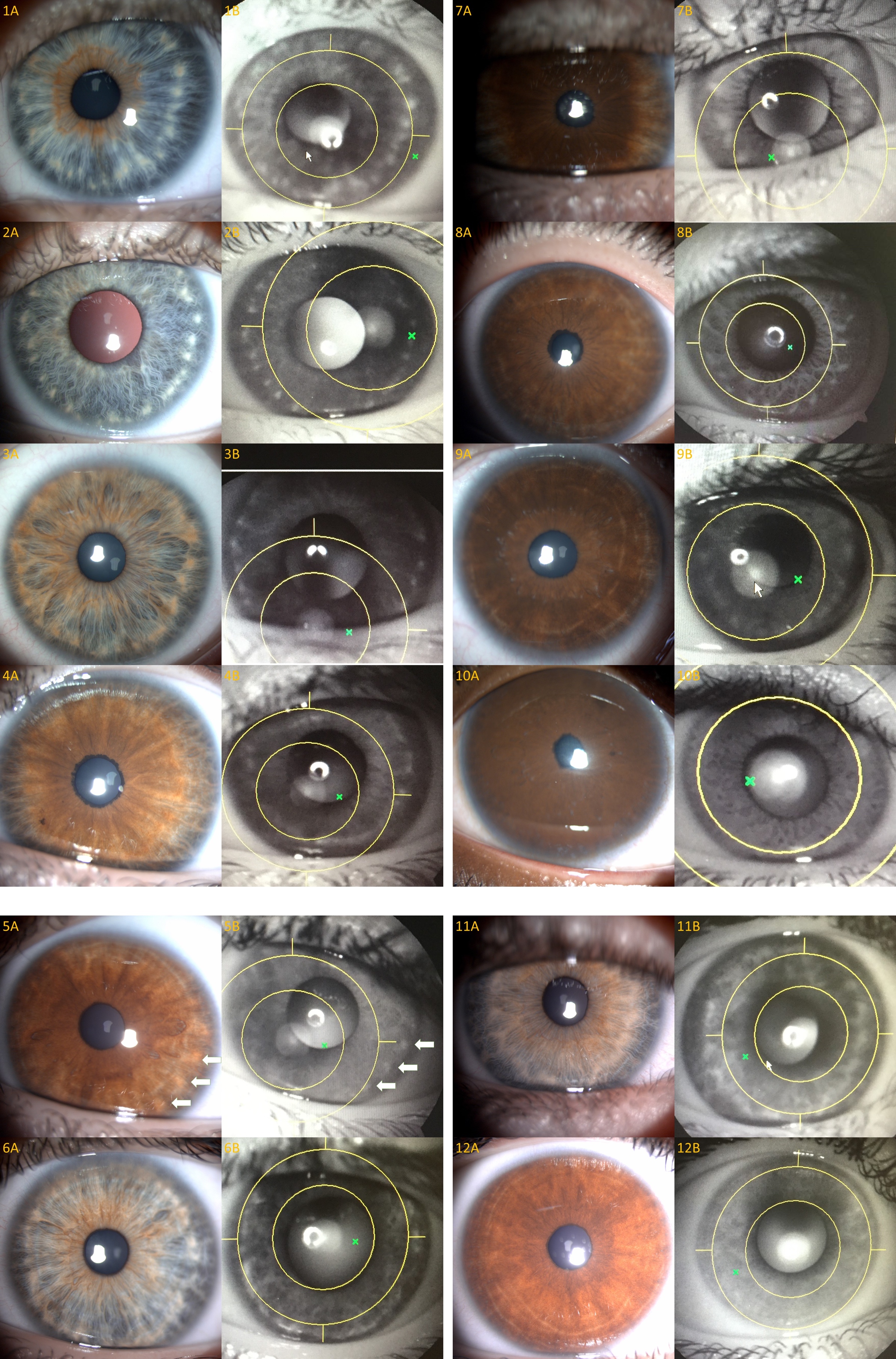

Supplement: Supplementary file 1 — Dataset 1 [file 41598_2018_36348_MOESM1_ESM.docx]
